# Yin and Yang in Post-Translational Modifications of Human D-Amino Acid Oxidase

**DOI:** 10.3389/fmolb.2021.684934

**Published:** 2021-05-10

**Authors:** Silvia Sacchi, Valentina Rabattoni, Matteo Miceli, Loredano Pollegioni

**Affiliations:** “The Protein Factory 2.0”, Dipartimento di Biotecnologie e Scienze della Vita, Università degli Studi Dell'Insubria, Varese, Italy

**Keywords:** D-serine, flavoprotein, nitrosylation, phosphorylation, biochemical properties, sulfhydration

## Abstract

In the central nervous system, the flavoprotein D-amino acid oxidase is responsible for catabolizing D-serine, the main endogenous coagonist of N-methyl-D-aspartate receptor. Dysregulation of D-serine brain levels in humans has been associated with neurodegenerative and psychiatric disorders. This D-amino acid is synthesized by the enzyme serine racemase, starting from the corresponding L-enantiomer, and degraded by both serine racemase (via an elimination reaction) and the flavoenzyme D-amino acid oxidase. To shed light on the role of human D-amino acid oxidase (hDAAO) in D-serine metabolism, the structural/functional relationships of this enzyme have been investigated in depth and several strategies aimed at controlling the enzymatic activity have been identified. Here, we focused on the effect of post-translational modifications: by using a combination of structural analyses, biochemical methods, and cellular studies, we investigated whether hDAAO is subjected to nitrosylation, sulfhydration, and phosphorylation. hDAAO is S-nitrosylated and this negatively affects its activity. In contrast, the hydrogen sulfide donor NaHS seems to alter the enzyme conformation, stabilizing a species with higher affinity for the flavin adenine dinucleotide cofactor and thus positively affecting enzymatic activity. Moreover, hDAAO is phosphorylated in cerebellum; however, the protein kinase involved is still unknown. Taken together, these findings indicate that D-serine levels can be also modulated by post-translational modifications of hDAAO as also known for the D-serine synthetic enzyme serine racemase.

## Introduction

The FAD-dependent enzyme D-amino acid oxidase (DAAO, EC 1.4.3.3) catalyzes the degradation of D-amino acids, the only exception being aspartate and glutamate (Pollegioni et al., 2007). In mammals, DAAO is mainly expressed in kidney, liver, brain, and gut (D'Aniello et al., 1993; Koga et al., 2017). In the brain, D-serine (D-Ser) represents the physiological substrate of DAAO. Indeed, D-Ser is the main endogenous coagonist of the N-methyl-D-aspartate type among the glutamate receptors (NMDAr): it binds the glycine site of these receptors, which regulate activity (Mothet et al., 2000) and physiological functions (Collingridge et al., 2013). Any dysregulation in the processes tuning D-Ser concentration alters NMDAr transmission and is thus involved in the mechanisms underlying various diseases of the central nervous system, such as amyotrophic lateral sclerosis, Alzheimer’s disease, schizophrenia, etc. (Collingridge et al., 2013; Paul and de Belleroche, 2014; Guercio and Panizzutti, 2018; Kondori et al., 2018; Uno and Coyle, 2019; Ploux et al., 2020). D-Ser metabolism in the brain is due to the activity of two enzymes: in addition to DAAO, serine racemase (SR, EC 5.1.1.18) catalyzes both the synthesis of D-Ser from the L-enantiomer and its degradation via an α,β-elimination reaction (Wolosker et al., 1999). In astrocytes, DAAO is the main enzyme involved in D-Ser catabolism since SR is only marginally expressed.

During the past few years, the structural-functional relationships in human DAAO (hDAAO) have been investigated using the recombinant protein produced in *E. coli* (Sacchi et al., 2012; Murtas et al., 2017; Pollegioni et al., 2018) and selected cell systems (Cappelletti et al., 2015; Murtas et al., 2019). Based on present knowledge, five strategies for regulating hDAAO have been identified:

(i) FAD binding: hDAAO possesses a weak interaction with FAD. The dissociation constant (K_d_) is in the micromolar range (Molla et al., 2006; Caldinelli et al., 2009), a value corresponding to the *in vivo* concentration of cofactor (Decker and Byerrum, 1954; Leighton et al., 1982). Accordingly, at conditions resembling physiological ones, hDAAO is present as an equilibrium between the apoprotein and the active holoenzyme;
(ii) substrate/ligand binding: the presence of a ligand in the active site promotes FAD binding (K_d_ decreases from 8 to 0.3 µM in the presence of 1 mM benzoate) (Molla et al., 2006; Caldinelli et al., 2010). We have suggested that substrate binding pushes its own degradation by increasing the amount of hDAAO active holoenzyme form in solution. Moreover, we recently demonstrated that hDAAO apoprotein exists in two alternative conformations differing in flavin cofactor affinity: the equilibrium is shifted toward the one at higher avidity by the presence of an active-site ligand (Murtas et al., 2017);
(iii) protein interaction: pLG72, bassoon, and PEX5 proteins have been reported to bind hDAAO (Sacchi et al., 2008; Popiolek et al., 2011; Sacchi et al., 2011; Maier et al., 2018). The primate-specific pLG72 protein in particular, appears to act as a negative chaperone of hDAAO, resulting in enzyme inactivation and higher instability (Sacchi et al., 2011). Notably, such an interaction has been related to schizophrenia susceptibility (Chumakov et al., 2002; Sacchi et al., 2016);
(iv) subcellular localization: cellular studies showed that part of the overexpressed hDAAO in U87 glioblastoma cells is cytosolic (Sacchi et al., 2011; Cappelletti et al., 2014). Indeed, recent reports on rats demonstrated that DAAO is present both in the cytosol and nuclei of proximal tubule epithelial cells following treatment with the drug propiverine (Luks et al., 2017b). A putative nuclear translocation signal has been identified in the primary sequence of hDAAO that could facilitate nuclear import via importin 7 after a TPx sequence has been phosphorylated (Luks et al., 2017a);
(v) post-translational modifications: it has been proposed that nitrosylation regulates hDAAO (Shoji et al., 2006) as its activity in U87 cells was enhanced by nitric oxide (NO) in a dose-dependent manner. The authors proposed that NO might accelerate D-Ser elimination in astrocytes by inhibiting SR and by activating hDAAO. Moreover, the observation that D-Cys represents the best substrate for the flavoenzyme (Murtas et al., 2017) and that DAAO and 3-mercapto-pyruvate sulfurtransferase are involved in generating H_2_S (Shibuya et al., 2013) suggests that H_2_S and/or sulfhydration might also affect hDAAO function.

Here, we focused on the modulation of hDAAO activity by three post-translational modification processes (nitrosylation, sulfhydration, and phosphorylation) with the aim to help elucidate the physiological functioning and alteration of this enzyme in pathological states.

## Materials and Methods

### Recombinant Proteins

The cDNA coding for hDAAO was cloned in the pET11b expression plasmid, under control of the T7 promoter. The His-tagged recombinant protein was expressed in *E. coli* BL21 (DE3) cells (Invitrogen, Carlsbad, CA, United States) upon induction with IPTG during the exponential phase of growth and purified by HiTrap chelating chromatography (GE Healthcare, Boston, MA, United States), as reported in (Murtas et al., 2017). For long storage at −80°C, the final protein preparation was equilibrated in 20 mM Tris–HCl buffer, 100 mM NaCl, pH 8.0, 10% glycerol, and 5 mM 2-mercaptoethanol, to which 40 μM FAD was added. The concentration of the purified enzyme was determined using the extinction coefficient at 455 nm (12.2 mM^−1^ cm^−1^) (Molla et al., 2006).

### Molecular Modeling and Structural Analyses

Solvent-accessible surface area analysis was performed on the hDAAO dimer structure (pdb code 2E49) using the algorithm provided by the Pymol open-source software (https://pymol.org/2/), which usually defines this parameter as the surface traced out by the center of a water sphere, having a certain radius rolled over the protein atoms; here, a default radius of about 1.4 Å was set.

### Cell Cultures

The U87 human glioblastoma cell line stably expressing hDAAO was generated by cloning the encoding cDNA in the pcDNA3 vector by restriction digestion: hDAAO cDNA was excised from pEYFP-hDAAO_C3 (Sacchi et al., 2011) using the HindIII and EcoRI sites. The resulting construct was confirmed by automated sequencing. U87 cells (ATCC, Manassas, VA, United States) were maintained in DMEM supplemented with 10% fetal bovine serum, 1 mM sodium pyruvate, 2 mM L-glutamine, penicillin/streptomycin, and amphotericin B (Euroclone S.p.A., Pero, Italy) at 37°C in a 5% CO_2_ (Sacchi et al., 2008). Transfection mixtures were set up using 6 μL of FuGENE HD transfection reagent (Promega Co., Madison, WI, United States) and 2 μg of the pcDNA3_hDAAO plasmid. Stable cell clones were selected by adding 0.4 mg/mL G418 to the growth medium. In the selected cell clones, hDAAO expression levels were monitored by Western blot analysis.

### Determination of Accessible Cysteines

To evaluate the number of accessible cysteines in hDAAO, the holo- and apoprotein forms were reacted with 0.1 mM DTNB in 0.45 M sodium phosphate buffer, pH 8.0, 0.45 mM EDTA, either under native conditions or in the presence of 4 M urea (Pollegioni et al., 1997). Samples were incubated for 15 min at room temperature and then the absorbance at 412 nm was recorded. Free sulfhydryl groups were quantified using the molar extinction coefficient of 2-nitro-5-thiobenzoic acid (TNB, ε_412 nm_ = 14,150 and 14,290 M^−1^cm^−1^ in sodium phosphate buffer and 4 M urea, respectively) and a calibration curve obtained using different concentrations of L-cysteine (0–200 μM).

### *In vitro* S-Nitrosylation

In order to ensure the long storage stability of recombinant purified enzyme, 2-mercaptoethanol was added to the storage buffer; the reducing agent was removed to perform *in vitro* modification studies. Thus, aliquots of hDAAO preparations were 10-fold diluted in nonreducing storage buffer (20 mM Tris–HCl pH 8.0, 100 mM NaCl, 10% glycerol, and 40 μM FAD) and concentrated to the starting volume using an Amicon centrifugal filter device (Ultracel–30K, Merck, Burlington, MA, United States). The procedure was repeated twice and protein concentration was determined spectrophotometrically.

*In vitro* nitrosylation was performed by incubating recombinant hDAAO with S-nitrosoglutathione (GSNO) as the NO donor (Wang et al., 2011). Reaction mixtures were prepared by diluting the protein in HEN buffer (25 mM HEPES, 1 mM EDTA, 0.1 mM neocuproine) to a final concentration of 1 mg/mL (25 µM) and adding 500 µM GSNO (200 µL final volume), in the presence or absence of 40 µM FAD. Mixtures containing 1 mg/mL BSA (reported to be S-nitrosylated by NO donors) (Stamler et al., 1992) or the recombinant protein to which 500 µM reduced glutathione (GSH) or oxidized glutathione (GSSG) was added were prepared as controls. Reaction mixtures were incubated 1 h in the dark, at 25°C, under constant rotation. S-Nitrosylation was verified by fluorescence detection using the fluorescence switch assay, following SDS-PAGE separation. The fluorescence switch assay was set up by using the fluorescent probe Alexa Fluor 350 C5 Maleimide (Molecular Probes/ThermoFisher Scientific, Waltham, MA, United States; excitation and emission wavelengths of 345 and 444 nm, respectively) and a procedure modified from (Han et al., 2008). The excess of GSNO (or GSH, or GSSG) was removed from the reaction mixtures by three steps of concentration/dilution in HEN buffer using Amicon Ultra 30 K 0.5 ml centrifugal filters (Merck). Unmodified free thiols were saturated by reacting the proteins with 20 mM MMTS in the presence of 2.5% SDS for 30 min at 50°C. MMTS was removed by 4 concentration/dilution steps in HEN buffer, as described above. Nitrosylated thiols were reduced by incubating the mixtures for 1 h at room temperature upon adding 5 mM sodium ascorbate and labeled with 50 µM Alexa Fluor 350 C5 Maleimide at 4°C for one night. Labeled samples were analyzed by SDS-PAGE under nonreducing conditions. The gel was rinsed with MilliQ water and imaged on a transilluminator using a Gel Doc 2000 (Biorad, Hercules, CA, United States). During the entire procedure samples were protected from light, in order to avoid dissociation of the modified thiols. Positive controls were prepared by omitting the MMTS blocking step, so that all cysteines were reduced during the incubation with sodium ascorbate.

### *In vitro* Sulfhydration

The modification of cysteine residues in hDAAO by H_2_S was investigated by incubating the recombinant protein with NaHS (Sigma-Aldrich, St. Louis, MO, United States) as a hydrogen sulfide donor. The protein samples were diluted in storage buffer without 2-mercaptoethanol to a concentration of 1 mg/mL, to which 1 mM NaHS was added, and incubated at 37°C for 1 h. The presence of modified cysteines was analyzed by performing the fluorescence switch assay following nonreducing SDS-PAGE, as detailed above.

### Cellular Nitrosylation and Sulfhydration Studies

The U87 stable cell clone ectopically expressing hDAAO was used to investigate the effect of nitrosylation and sulfhydration on enzyme cellular activity, while cells transfected with the empty pcDNA3 vector were used as controls. Cells were maintained in T75 flasks; when the cell density reached ∼80% of confluence, they were washed in PBS and 10 mL of serum free DMEM containing 50 µM of either NOC7 or NOR3 as NO donors, or a corresponding amount of vehicle, was added (Santa Cruz Biotechnology, Dallas, TX, United States) and they were placed back in 5% CO_2_ at 37°C for 2 h. Treated and control cells were then collected by trypsinization, centrifuged, washed rapidly with ice-cold PBS, and assayed for hDAAO activity. Lysates were prepared by suspending treated and control cells in 50 mM sodium phosphate, pH 7.0, 0.7 μg/mL pepstatin, 1 μg/mL leupeptin, 10 µM FAD, and 0.1% ethanol and then sonicating the cell suspension (3 cycles, 10 s each, intersperse with 30 s in ice). Samples were clarified by centrifugation (13,000 × g, for 15 min at 4°C), and total protein content was quantified by using the Bradford reagent (Sigma-Aldrich). Amounts of treated and control cell lysates corresponding to 50 µg of total proteins were used to measure hDAAO activity, on a 96-well plate by using the Amplex UltraRed assay as previously reported (Sacchi et al., 2011), see Activity assays section, below.

The same procedure was used to assess the effect of sulfhydration on hDAAO cellular activity. In this case, cells were treated for 30 min with 50 µM NaHS, as a hydrogen sulfide donor.

### *In vitro* Phosphorylation

Reaction mixtures were prepared in a final volume of 50 µL using three protein kinases. The PKA reaction mixture contained 3.4 µM (6.8 µg, 0.17 nmoles) of recombinant hDAAO in 50 mM Tris, pH 7.5, 50 µM ATP, 10 mM MgCl_2_, and 1 mM DTT. Alternatively to hDAAO, 4 µg of the recombinant GST-tagged CREB1 (Merk Millipore; ∼ 59 kDa, 0.11 nmoles, 2.2 µM) was used as a positive control (Gonzalez and Montminy, 1989). Then, 0.009 U of recombinant PKA (Merck) was diluted in 50 mM HEPES, pH 7.4, 1 mM DTT, and added to each assay mixture to start the reaction. Analogously, PKC-α and PKC-ε (Ab Cam, Cambridge, United Kingdom) reaction mixtures contained 6.8 µg of recombinant hDAAO in 20 mM HEPES, pH 7.4, 50 µM ATP, 10 mM MgCl_2_, 1 mM CaCl_2_, 0.5 mg/mL phosphatidylserine, 0.05 mg/mL diacylglycerol, 1 mM sodium orthovanadate, and 1 mM DTT. In these cases, positive controls used the histone H1 (Sigma-Aldrich, 4 μg, 0.19 nmoles, 3.8 µM) (Zhao et al., 2004). The recombinant PKC-α and PKC-ε were diluted in 65 mM Tris-HCl, pH 7.5, 0.87% NaCl (w/v), 2 mM DTT; 0.001 U of PKC-α or 0.0005 U of PKC-ε was added to start the reaction. All reactions were carried out at 30°C and sample aliquots (15 μL, 2 µg hDAAO) were collected at time 0, 2 h, and overnight and then blocked by adding 4X Laemmli sample buffer (5 µL) and boiling. The different samples were resolved by SDS-PAGE and phosphorylated proteins detected by using the Pro-Q Diamond Phosphoprotein Gel Stain (Thermo Fisher Scientific), following the procedure indicated by the supplier. As a staining control, a molecular ladder (PeppermintStick Phosphoprotein Molecular Weight Standards, Thermo Fisher Scientific) containing phosphorylated (45.0 and 23.6 kDa) and not phosphorylated (116, 66.2, 18.0, and 14.4 kDa) proteins was used. Gels were imaged using an Odissey Fc imaging system (LI-COR Biotechnology, Lincoln, United States) and the 600-nm channel with a light source for excitation.

### Tissue Analysis of Human D-Amino Acid Oxidase Phosphorylation Levels

The hDAAO endogenously present in human brain tissues (cerebellum and prefrontal cortex obtained from the London Neurodegenerative disease Brain Bank, King’s College, London, United Kingdom) was isolated by immunoprecipitation (IP). Tissue samples were from a healthy, male subject, 58 years of age (PMD 20 h). Briefly, 100–120 mg were resuspended in lysis buffer: 20 mM Tris-HCl, pH 8.0, 150 mM NaCl, 0.5% NP-40, 1 mM EDTA, 2 μM leupeptin, 1 μM pepstatin, 500 μM PMSF, and phosphatase Inhibitor Cocktail (Cell Signaling Technologies, Danvers, MA, United States), homogenized with a microcentrifuge tube potter, and sonicated (3 cycles, 20 s each, interspersed with 1-min incubation in ice). Lysates were clarified by centrifugation (13,000 × g for 30 min at 4°C) and the total protein concentration in the supernatants (Pre-IP samples) was quantified using the Bradford assay (Sigma-Aldrich). Equivalent amounts of samples (1.5 mg total proteins) were subjected to immunoprecipitation, which was performed by crosslinking rabbit polyclonal anti-hDAAO antibodies (10 μg, Davids Biotechnologie, Regensburg, Germany) to Dynabeads Protein G (Invitrogen, Carlsbad, CA, United States) using 20 mM dimethyl pimelimidate dissolved in 0.2 M triethanolamine, pH 8.2 (30 min at room temperature under rotation). Negative controls were set up by omitting the antibodies in the crosslinking reaction. After removing the excess of crosslinker, the antibody-crosslinked Dynabeads were incubated with the lysate samples overnight at 4°C, under rotation. The supernatants (post-IP samples) were collected by separating the beads on the magnet, which were then extensively washed with lysis buffer and finally suspended in 55 μL of a nonreducing 1X Laemmli sample buffer (IP sample) and boiled. To confirm the presence of hDAAO, 10 μL of the IP samples were analyzed by Western blotting, while the remaining samples (40 μL) were resolved by SDS-PAGE and imaged using the Pro-Q Diamond Gel Stain to detect the phosphorylation levels of the immunoprecipitated hDAAO.

Western blot analyses were performed using the anti-hDAAO primary antibody (1:1,000, Davids Biotecnologie) and a peroxidase-conjugated donkey anti-rabbit IgG secondary antibody (1:15,000, Jackson ImmunoResearch, Ely, United Kingdom).

### Activity Assays

Following *in vitro* nitrosylation and sulfhydration, hDAAO activity was assayed polarographically using an oxygen electrode (Molla et al., 2006). Aliquots withdrawn from the reaction mixtures were diluted in the storage buffer without 2-mercaptoethanol (1:10) and immediately assayed for residual activity with an oxygen electrode at pH 8.5, air saturation, and 25°C, using 28 mM D-alanine as substrate in the presence of 0.2 mM FAD. Control measurements were performed in the absence of NO or H_2_S donors. The residual enzymatic activity was calculated by comparing the values measured before and after performing the *in vitro* modification reactions. The statistical significance of data was assessed by means of an unpaired *t*-test, *n* = 5.

The effect of either nitrosylation or sulfhydration on hDAAO functionality was also investigated at the cellular level. U87 cells ectopically expressing hDAAO were treated with the NO donors NOR-3 (50 µM for 2 h), as reported in (Shoji et al., 2006) or NOC7 (50 µM for 2 h), or with the hydrogen sulfide donor NaHS (100 µM for 30 min), or an equal amount of vehicle (DMSO and H_2_O, respectively). Upon treatments, the enzyme activity was assayed on cell lysates by using the Amplex UltraRed reagent (Invitrogen, Carlsbad, CA, United States) as reported in (Sacchi et al., 2011). The reaction was started by diluting cell lysates 1:2 in the activity assay solution containing 50 μM Amplex UltraRed, 0.2 U/ml horseradish peroxidase, 10 mM NaN_3_, 10 μM FAD, and 50 mM D-Ser in 50 mM sodium phosphate, pH 7.0. The assay is based on the detection of H_2_O_2_ by peroxidase-mediated oxidation of the fluorogenic Amplex UltraRed Dye. Negative controls were performed using the activity assay solution lacking the substrate D-Ser. hDAAO activity was determined by monitoring the fluorescence emission at 590 nm (upon excitation at 535 nm) over time (50 min) and comparing the difference in fluorescence emission between samples (from cells treated with the NO or H_2_S donors) and control assay mixtures. U87 cells transfected with the pcDNA3 empty vector were used as further control. Data significance was evaluated by means of a two-way analysis of variance (ANOVA) and a multiple *t*-test on grouped measures: 4 measurements were performed at each time point and the experiment was repeated twice.

### Spectral Measurements

hDAAO apoprotein was prepared by overnight dialysis of the holoenzyme against 1 M KBr as reported in (Murtas et al., 2019) and the final concentration was determined based on the extinction coefficient at 280 nm (75.2 mM^−1^ cm^−1^). Fluorescence spectra were measured at 1 μM protein concentration (0.04 mg/mL) in 50 mM sodium pyrophosphate, pH 8.3, and 5% glycerol; spectra were recorded using a Jasco FP-750 instrument and corrected for the buffer contribution. Protein fluorescence spectra were recorded between 300 and 400 nm, with excitation at 280 nm.

The ligand dissociation constants were estimated by titrating the apoprotein with increasing amounts of FAD and following the quenching of protein fluorescence at 330–340 nm (Molla et al., 2006). The K_d,FAD_ values were determined in the presence of different concentrations of the NO donor GSNO or the H_2_S donor NaHS. *In vitro* nitrosylation mixtures containing hDAAO apoprotein (1 mg/mL, 25 μM) were prepared in 100 μL 50 mM sodium pyrophosphate pH 8.3, 5% glycerol, 0.1 mM neocuproine, and 50 or 500 μM GSNO and incubated for 1 h in the dark, at 25°C, under constant rotation. Mixtures were then diluted 25-fold in the same buffer (1 μM apoprotein final concentration) and transferred to the cuvette for titration with FAD. To assess the effect of the hydrogen sulfide donor, mixtures containing hDAAO apoprotein (1 μM) were set up in 20 mM Tris–HCl buffer, 100 mM NaCl, pH 8.0, and 10% glycerol to which 5 or 40 μM NaHS was added and incubated 30 min at room temperature before titration. Control experiments were performed by omitting the modifying agents. In all cases, K_d,FAD_ values were estimated by hyperbolic interpolation of the experimental data (Murtas et al., 2017).

## Results

### Prediction of Secondary Modification Sites in hDAAO

**FIGURE 1:**
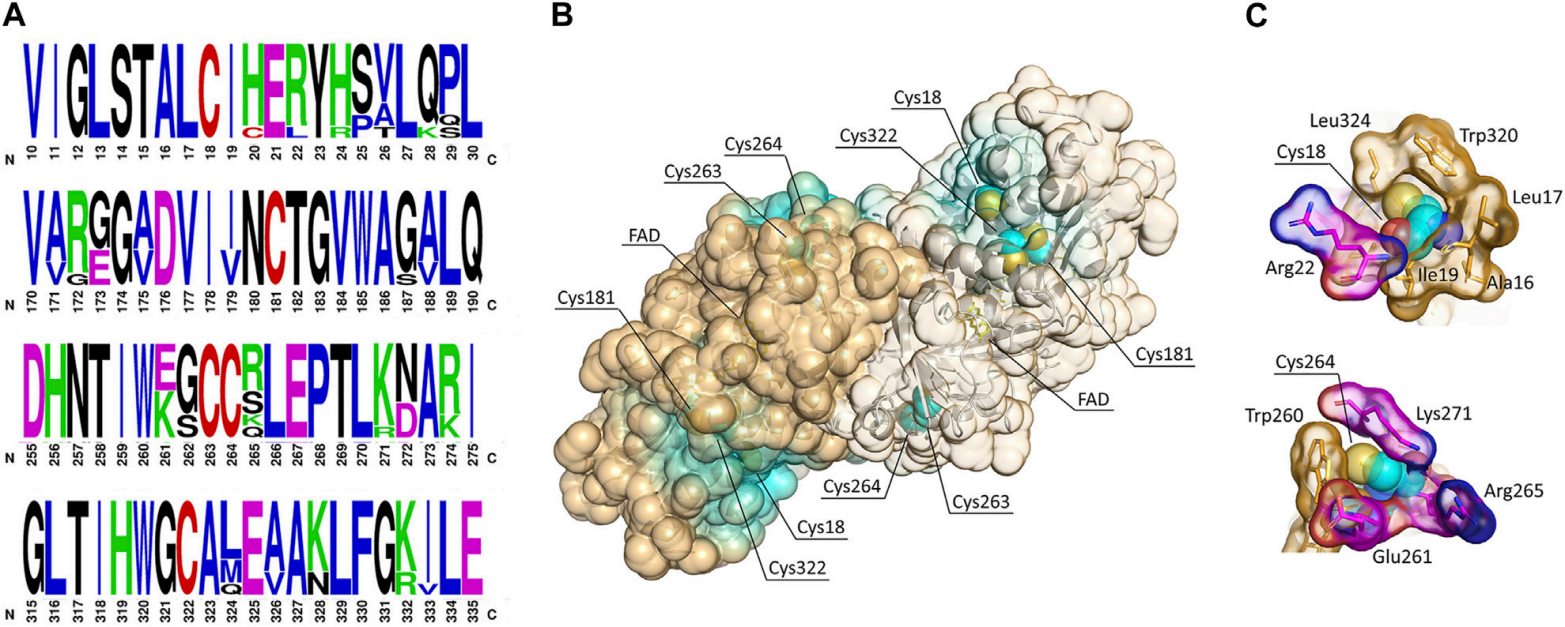
Cysteine residues in hDAAO. **(A)** Weblogo representation of conserved residues identified by the alignment of DAAO sequences from *Homo sapiens, Mus musculus, Rattus norvegicus, Sus scrofa, Bos taurus, Cavia porcellus,* and *Macaca fascicularis*. The *x*-axis represents amino acid positions (the annotated numbering refers to the human enzyme). The height of symbols is proportional to the degree of conservation of single residues. Panels represent sequence stretches of 20 residues containing cysteine residues, shown in red. Five cysteine residues appear highly conserved in mammalian DAAOs. Figure prepared using WebLogo (https://weblogo.berkeley.edu/logo.cgi). **(B)** Solvent-accessible surface (SAS) as calculated for the hDAAO dimer (pdb code 2E49). Cysteine residues are represented as sphere (carbon atoms in cyan, sulfur atoms in yellow), the FAD cofactor is represented as sticks (yellow), and the backbone is shown as cartoon. Protein surface is colored by proximity of cysteine residues (cyan = cysteine residues located within 5 Å from the surface; orange = cysteine residues located at a distance higher than 15 Å from the surface). SAS has been calculated based on a solvent radius of 1.4 Å. Two of the conserved cysteine residues are likely accessible to modifications. **(C)** Details showing the amino acidic environment of selected cysteine residues, represented as spheres (carbon atoms in cyan, sulfur atoms in yellow, nitrogen atoms in blue, and oxygen atoms in red). The surrounding residues are depicted as sticks; the van der Waals surface is shown. Hydrophobic residues are colored in orange, while charged residues are colored by element (carbon atoms in magenta, nitrogen atoms in blue, and oxygen atoms in red). Illustrations prepared with “The Protein Imager online molecular viewer” (https://3dproteinimaging.com/protein-imager/).

Although NO has been proposed to positively affect hDAAO activity in U87 human glioblastoma cells in a dose-dependent manner (Shoji et al., 2006), the observed effect has not been investigated further, nor has the modification of cysteine residues by S-nitrosylation been evaluated. hDAAO contains five cysteines residues (Figure 1). Four cysteines are conserved in both hDAAO and the homologous flavoenzyme human d-aspartate oxidase (containing a total of nine cysteines).

Putative nitrosylated residues in hDAAO were predicted by using different web-based tools (Table 1): these analyses yielded divergent results. It is noteworthy that the web-based tool SNOsite (Lee et al., 2011), which applies the maximal dependence decomposition approach to explore conserved nitrosylation motifs, identified the totality of hDAAO cysteines as potential nitrosylation sites (Table 1). In native hDAAO, all cysteine residues should be present in the free reduced form as no disulfide bonds were apparent in the crystal structure (pdb 2E49) and have been identified in the -SH state by the disulfide bonding-state predictors DISULFIND at http://disulfind.dsi.unifi.it/ (Ceroni et al., 2006) and CYSPRED at http://gpcr.biocomp.unibo.it/cgi/predictors/cyspred/pred_cyspredcgi.cgi (Fariselli et al., 1999).

**TABLE 1:** Prediction of nitrosylation sites by using web-based tools. Cysteine residues indicated in bold are strictly conserved in hDAAO. In the last column, underscored residues are those present in the consensus sequence of putative nitrosylation motifs as identified by the SNO site.

Cys position	GPS	iSNO	iSNO	Deep	SNO	Sequence
	SNO 1.0	PseAAC	AAPair	Nitro	Site	−10+10
18			●		●	AGVIGLSTAL**C**IHERYHSVLQ
181	●				●	AREGADVIVN**C**TGVWAGALQR
263					●	IQDHNTIWEG**C**CRLEPTLKNA
264		●			●	QDHNTIWEGC**C**RLEPTLKNAR
322			●		●	GGYGLTIHWG**C**ALEAAKLFGR

GPS SNO 1.0 at http://sno.biocuckoo.org (Xue et al., 2010); iSNO PseAAC at http://app.aporc.org/iSNO-PseAAC/(Xu et al., 2013a); iSNO AAPair at http://app.aporc.org/iSNO-AAPair/(Xu et al., 2013b); DeepNitro at http://deepnitro.renlab.org/webserver.html (Xie et al., 2018); SNOSite at http://csb.cse.yzu.edu.tw/SNOSite/Prediction.html (Lee et al., 2011).

For protein S-sulfhydration (Mustafa et al., 2009), it is currently not feasible to predict putative modification sites, although accumulated evidence suggests that this modification may share chemical features with protein S-nitrosylation.

Phosphorylation sites were also predicted by means of computational tools (Table 2). Although several residues in hDAAO were identified as “modified” by the different predictors, the estimated levels of serine, threonine, and tyrosine phosphorylation were heterogeneous. Moreover, in the case of DIPHOSPH 1.3, no phosphorylation site was identified when the group predictor for human was selected instead of the default predictor. Nonetheless, hDAAO phosphorylation at Tyr23 is documented in PhosphoSitePlus (https://www.phosphosite.org). When single kinases are specified as the putative modifying enzyme, hDAAO appeared to be mainly subjected to phosphorylation by cAMP-dependent protein kinase (PKA), protein kinase C (PKC), and protein kinases involved in cell cycle regulation (cdk5 and CKII).

**TABLE 2:** Prediction of phosphorylation sites in hDAAO by using web-based tools. For netPhos 3.0 prediction, the score value was set above 0.75.

Residues (total number)	NetPhos 3.0	DIPHOSPH 1.3	PHOSPHONET	GPS 5.0
Ser (15)	4 (27%)	2 (13%)	7 (47%)	−
Thr (22)	5 (22%)	4 (18%)	8 (36%)	−
Tyr (12)	2 (17%)	1 (8%)	8 (67%)	−

NetPhos 3.0 at http://www.cbs.dtu.dk/services/NetPhos/(Blom et al., 1999); DIPHOSPH 1.3 at http://www.dabi.temple.edu/disphos/; PHOSPHONET at http://www.phosphonet.ca; and GPS 5.0 at http://gps.biocuckoo.cn (Wang et al., 2020).

### Two Cysteines are Solvent Accessible in hDAAO

The extent to which an amino acid interacts with the solvent and the protein core is proportional to the degree of exposition to these environments. The solvent-accessible surface area (SAS) is a geometric measure of this exposure, and therefore a correlation exists between SAS and environment-free energy (Pokala and Handel, 2004; Vizcarra and Mayo, 2005). The exposure of cysteines to modification was assessed by performing a solvent-accessible surface analysis on the protein structure (Figure 1B). In hDAAO, Cys18 and Cys264 appear to be exposed on the protein surface, while the other three cysteine residues point toward the interior of the protein structure and thus are less likely accessible to modification (Figure 1B). Notably, Cys18 is surrounded by hydrophobic residues (Figure 1C, top) and is located at 8.2 Å from Arg22, which is part of a putative nitrosylation motif consensus sequence (Table 1): these two features might stabilize the S-nitrosylated cysteine residues (SNO-Cys) once modified (Marino and Gladyshev, 2010). Worthy of note is that hDAAO cysteines appear to be grouped in small clusters (Figure 1B).

The solvent accessibility of cysteine residues was experimentally evaluated by reacting the recombinant hDAAO with 5,5′-dithiobis (2-nitrobenzoic acid) (DTNB) under nonreducing conditions and in the absence or presence of a denaturing agent. Consistent with structural information, the reaction of native hDAAO with DTNB yielded 1.85 ± 0.2 free cysteines per protein monomer, whereas in the presence of 4 M urea the assay yielded a number of 5.9 ± 0.6, indicating that three cysteines are not solvent accessible in the native conformation and confirming that no disulfide bonds are present. Notably, under native conditions the apoprotein and the holoenzyme forms of hDAAO exposed the same number of Cys (1.9 ± 0.2).

### Human D-Amino Acid Oxidase S-Nitrosylation Studies

**FIGURE 2:**
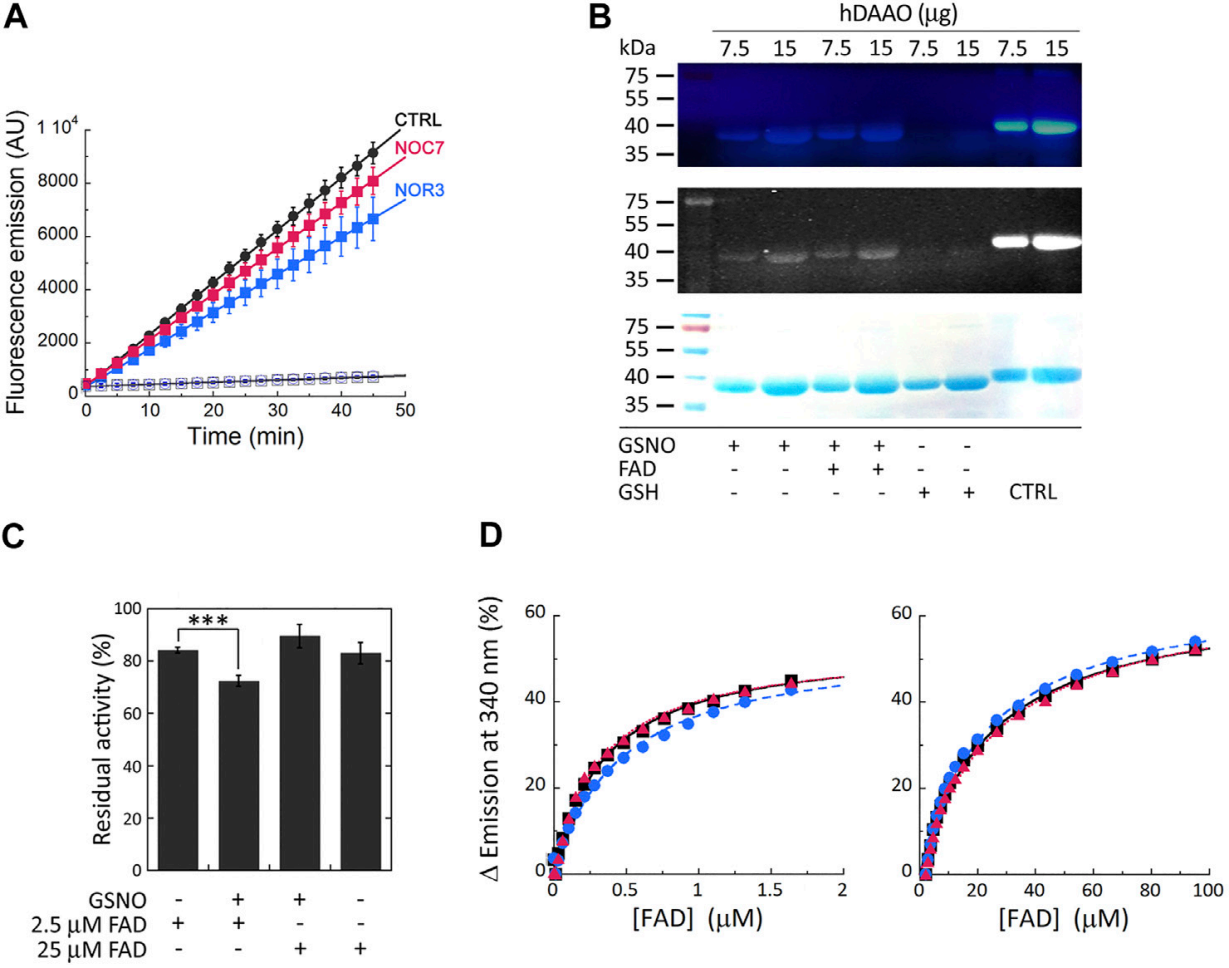
S-Nitrosylation of hDAAO induces partial inactivation. **(A)** The treatment of U87 cells ectopically expressing hDAAO with the NO donors NOR3 and NOC7 reduced the activity of the flavoenzyme. hDAAO-expressing cells were treated with 50 µM NOC7 (red squares), 50 µM NOR3 (blue squares), or with an equal amount of DMSO (CTRL, black circles) for 2 h. Cells transfected with the pcDNA3 empty vector and treated with the same amounts of the NO donors (blue open circles) or DMSO (black open square) were used as further controls. In treated and control cells hDAAO activity was detected in cell lysates by the Amplex UltraRed assay and reported as fluorescence emission at 590 nm over time. Values are reported as mean ± SEM, *n* = 5. **(B)** hDAAO is nitrosylated *in vitro*. The nonreducing SDS-PAGE analysis of recombinant hDAAO (7.5 and 15 µg) following *in vitro* S-nitrosylation and the fluorescence switch assay is shown. Mixtures in which the NO donor GSNO was replaced with GSH were analyzed as negative controls whereas the positive controls (CTRL) were represented by protein samples in which all cysteine residues were labeled by the Alexa Fluor 350 C_5_ Maleimide reagent (by omitting the MMTS blocking step during the fluorescence switch assay). Image acquisition was performed by a normal **(top panel)** and a CCD **(middle panel)** camera upon excitation of the fluorescent probe. Proteins were also stained with Coomassie blue **(bottom panel)**. **(C, D)** Nitrosylation slightly alters hDAAO enzymatic stability without affecting the binding of the FAD cofactor. **(C)** Effect of the presence of GSNO on hDAAO activity, as determined by the oxygen consumption activity assay. *In vitro* nitrosylation mixtures and related controls were set up in the absence or presence of exogenous FAD (2.5 and 25 µM final concentration). Residual activity values (100% represents the initial activity of the unreacted enzyme) are reported as mean ± SEM, *n* = 5, ***p < 0.005, unpaired *t*-test. **(D)** Analysis of FAD binding to hDAAO apoprotein in the absence and presence of 50 or 500 µM of the NO donor GSNO, assessed as protein fluorescence quenching. Values are expressed as percentage of the total change in a single experiment (the determined K_d_ values are the average of three independent experiments). Left panel shows the interpolation of the experimental data corresponding to the first saturation phase of the protein fluorescence change (up to 2 µM of cofactor concentration); right panel reports the same analysis for the second phase of saturation. On the *y*-axis, 100% correspond to the change in fluorescence emission between the hDAAO apoprotein free form and the fully FAD-complexed one. Measurements were performed at 15°C. Black squares and continuous lines: control mixture in the absence of GSNO; blue circles and staggered lines: mixture containing 50 µM GSNO; red triangles and dotted lines: mixture containing 500 µM GSNO.

Cellular studies were performed on a U87 stable cell clone ectopically expressing hDAAO: cells were treated with the membrane-permeable NO donors NOC7 and NOR3 and activity assays were performed on the cell lysates using the fluorescent Amplex UltraRed reagent. Compared to control cells, significantly decreased fluorescence emission values were detected when cells were treated with NO donors (especially NOR3; treatment × time interaction F (18, 108) = 29.47, *p* < 0.0001 and F (18, 108) = 11.8, *p* < 0.0001 for NOR3 and NOC7, respectively), while no signal changes were detected for lysates from cells transfected with the empty pcDNA3 vector, confirming the specificity of the assay (Figure 2A).

*In vitro* nitrosylation reaction mixtures were set up using pure, recombinant hDAAO solutions (25 μM, depleted of the reducing agent 2-mercaptoethanol) and 500 µM S-nitrosoglutathione (GSNO) as a NO donor (or reduced/oxidized glutathione as negative controls). The reaction was also performed after adding 40 µM FAD to push the equilibrium in solution between the apoprotein and the holoenzyme forms of hDAAO toward the latter form. Modified SNO-Cys residues were detected by a fluorescent switch assay in which the SNO-Cys residues were selectively labeled with Alexa Fluor 350 C5 Maleimide. SDS-PAGE analysis performed under nonreducing conditions clearly indicated that GSNO modified hDAAO (Figure 2B). No aspecific labeling was observed in the negative control samples. The presence of exogenous FAD in the S-nitrosylation mixtures did not affect the extent of hDAAO modification, as a similar signal intensity was apparent for the bands corresponding to the protein incubated with GSNO in the presence or absence of the flavin cofactor. The latter observation agrees with the results from DTNB assay, indicating that two cysteines were modified for both the hDAAO apoprotein and holoenzyme forms.

The effect of S-nitrosylation was investigated *in vitro* by measuring the residual enzymatic activity of recombinant hDAAO upon incubation with the NO donor at two concentrations of FAD corresponding to 25 µM (i.e., equimolar to hDAAO concentration) and 2.5 µM. The latter concentration was selected to reproduce physiological conditions (Decker and Byerrum, 1954; Leighton et al., 1982). A slight, but statistically significant, reduced hDAAO activity was observed in the presence of 2.5 µM FAD compared to 25 µM FAD or control conditions (Figure 2C, residual acitivity 72 ± 2, 89 ± 4 and 84 ± 2, respectively, *p* < 0.005). The protective effect of an equimolar amount of the cofactor suggests that the cysteine residue(s) subjected to S-nitrosylation are likely more reactive in the hDAAO apoprotein form. Spectral experiments monitoring protein fluorescence quenching during titration of the hDAAO apoprotein with increasing concentrations of FAD in the presence of 50 or 500 µM of the NO donor showed no change in K_d_ values for the apoprotein-FAD complex or in the equilibrium between the two known apoprotein conformations (Murtas et al., 2017) (Figure 2D; Table 3). This result excludes the notion that nitrosylation may affect the interaction of the cofactor with the apoprotein moiety while it does suggest that nitrosylation results in an alteration in hDAAO conformation that partially inactivates the enzyme, a process that is faster for the apoprotein form than for the holoenzyme counterpart.

**TABLE 3:** Effect of nitrosylation or sulfhydration on the FAD-apoprotein hDAAO complex. The cofactor dissociation constants (K_d_) determined by measuring the protein fluorescence quenching during the titration of the apoprotein with FAD in the absence or presence of different concentrations of the NO donor GSNO or of the H_2_S donor NaHS, under different buffer conditions.

	GSNO (µM)			NaHS (µM)		
	0	50	500	0	5	40
First phase						
K_d_ (µM)	0.35 ± 0.02	0.34 ± 0.04	0.31 ± 0.02	0.43 ± 0.02	0.31 ± 0.03	0.33 ± 0.02
Amplitude (%)	45	46	44	42	50	48
Second phase						
K_d_ (µM)	24.2 ± 2.1	23.0 ± 2.0	26.7 ± 2.3	≥30	27.5 ± 3.2	27.8 ± 3.2
Amplitude (%)	55	54	56	58	50	52

### Human D-Amino Acid Oxidase Sulfhydration Studies

**FIGURE 3:**
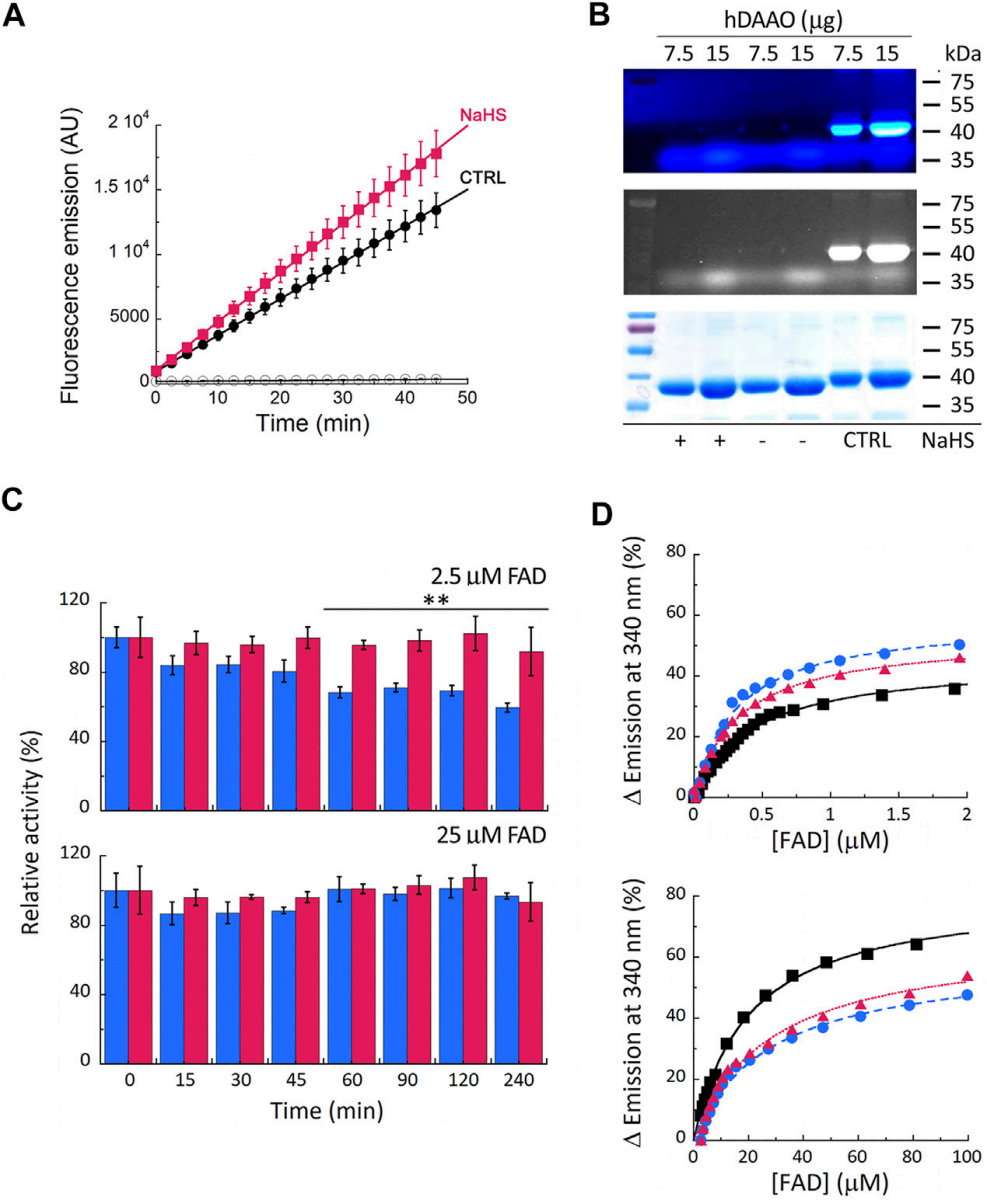
Effect of S-sulfhydration on hDAAO properties. **(A)** Treatment of U87 transfected cells expressing hDAAO with the hydrogen sulfide donor NaHS increases the activity of the flavoenzyme. Cells were treated with 50 µM NaHS (red squares) or with an equal amount of H_2_O (CTRL, black circles) for 30 min. Cells transfected with the pcDNA3 empty vector and treated with the same amount of the H_2_S donor (open circles) were used as further controls. In treated and control cells hDAAO activity was detected in cell lysates by the Amplex UltraRed assay and reported as fluorescence emission at 590 nm over time (0–45 min). Values are reported as mean ± SEM, *n* = 5. **(B)** hDAAO is not sulfhydrated *in vitro*. The nonreducing SDS-PAGE analysis of recombinant hDAAO (7.5 and 15 µg) following *in vitro* S-sulfhydration and the fluorescence switch assay is shown. Mixtures in which the hydrogen sulfide donor NaHS was omitted were analyzed as negative controls whereas positive controls (CTRL) were represented by protein samples in which all cysteine residues were labeled by Alexa Fluor 350 C_5_ Maleimide (see legend of Figure 2B for details). Proteins were also stained with Coomassie blue **(bottom panel)**. **(C, D)** The hydrogen sulfide donor NaHS appears to stabilize recombinant hDAAO and affect the FAD cofactor binding. **(C)** Effect of 1 mM NaHS on the time course of hDAAO activity, as determined by the oxygen consumption activity assay. *In vitro* sulfhydration mixtures (red bars) and related controls (blue bars) were set up in the absence or presence of exogenous FAD (2.5 or 25 µM final concentration). Residual activity values (100% represents the initial activity of the unreacted enzyme) are reported as mean ± SEM, *n* = 5, **p < 0.005, unpaired *t*-test. **(D)** Analysis of FAD binding to hDAAO apoprotein in the absence and presence of 5 or 40 µM of the hydrogen sulfide donor NaHS, assessed as protein fluorescence quenching. Values are expressed as percentage of the total change. Top panel shows the interpolation of the experimental data corresponding to the first saturation phase of the protein fluorescence change (up to 2 µM of cofactor concentration); bottom panel reports the same analysis for the second phase of saturation. Measurements were performed at 15°C. Black squares and continuous lines: control mixture in the absence of NaHS; blue circles and staggered lines: mixture containing 5 µM NaHS; red triangles and dotted lines: mixture containing 40 µM NaHS.

Cellular studies on U87 cells ectopically expressing hDAAO showed that the enzyme activity significantly increases in cells treated with the hydrogen sulfide donor NaHS compared to controls (Figure 3A, treatment × time interaction F (18, 90) = 4.331, *p* < 0.0001). *In vitro* sulfhydration reaction mixtures were set up by diluting the recombinant hDAAO (25 μM, depleted of the reducing agent), in nonreducing storage buffer added with 40 µM FAD: no signal due to protein modification was apparent (Figure 3B), suggesting that hDAAO sulfhydration, if there is any, is below the detection limit.

The incubation of hDAAO with the hydrogen sulfide donor in the presence of a low concentration of exogenous FAD (2.5 µM, molar ratio hDAAO:FAD = 1:1) affected flavoenzyme stability (Figure 3C, top panel): in the control mixture (i.e., in the absence of NaHS), residual activity decreased to 59 ± 3% after 4 h of incubation at 37°C, while it was unchanged in the mixture containing 1 mM NaHS (92 ± 14%). Notably, the same stabilizing effect was obtained by adding an excess of free FAD (25 μM, molar ratio hDAAO:FAD = 1:10) instead of NaHS. Differently from GSNO, the hydrogen sulfide donor affected FAD binding to hDAAO apoprotein (Figure 3D): the equilibrium between the two apoprotein conformations is slightly altered by NaHS, and the K_d_ values for FAD binding are decreased for both phases (Table 3). Notably, NaHS concentrations in the low µM range are sufficient to induce the observed effect: the tighter hDAAO-FAD interaction observed in the presence of 5 µM NaHS increases the amount of active holoenzyme form in solution, thus explaining the observed increase in both enzymatic activity (Figure 3A) and stability (Figure 3C).

### Human D-Amino Acid Oxidase Phosphorylation Studies

**FIGURE 4:**
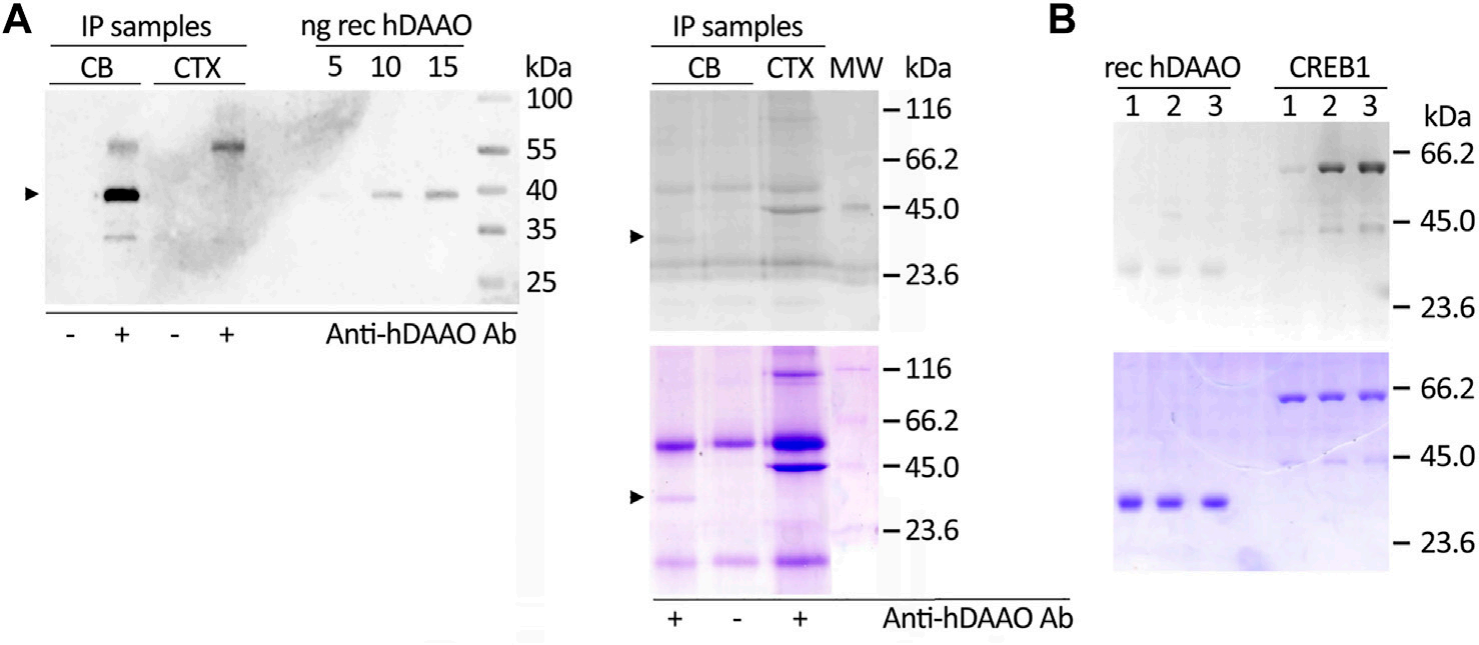
Phosphorylation of hDAAO. **(A)** In the cerebellum hDAAO is phosphorylated. **Left)** The flavoenzyme was immunoprecipitated from cerebellum lysates (CB) but not from cortex samples (CTX), as verified by Western blot. **Right)** The flavoenzyme phosphorylation levels were detected by the Pro-Q Diamond Gel Stain **(top panel)** and the identity of the observed 40 kDa bands further verified by Coomassie blue staining **(bottom panel)**; in both panels arrowheads indicate the corresponding hDAAO band. Immunoprecipitations performed either on cerebellum lysates in the absence of hDAAO antibodies or on cortex lysates (where the protein is present at trace levels) represented negative controls while diluted PeppermintStick Phosphoprotein Molecular Weight Standards (0.125 µg each protein, MW lane) were used as positive controls. The additional faint bands (at ∼ 55 and 30 kDa) observed in the left panel should correspond to the anti-hDAAO IgG heavy chain and to protein G, respectively. The bands present in right panel, in addition to the 40 kDa one corresponding to hDAAO, are aspecific signals due to proteins interacting with the beads regardless of the presence of the crosslinked antibody. **(B)** *In vitro* phosphorylation experiments indicated that hDAAO is not modified by PKA. SDS-PAGE analysis of aliquots of the reaction mixtures containing 2 µg of recombinant hDAAO or 1.2 µg GST-tagged CREB1 as a positive control, collected at different times of incubation at 30°C (1 = 0 min; 2 = 2 h; 3 = overnight). Upon resolution on a 12% acrylamide gel, phosphoproteins were detected by the Pro-Q Diamond Gel Stain **(top panel)**: while the 59-kDa band corresponding to CREB1 was positively stained, no detectable signal was apparent for the 40-kDa band corresponding to hDAAO. The amount of loaded proteins was verified by subsequently staining the gel in Coomassie blue **(bottom panel)**.

As hDAAO was predicted to be phosphorylated (likely by PKA and/or PKC, among other kinases, see above), we analyzed the protein endogenously expressed in human brain tissues using cerebellum lysates (expected to be strongly enriched in hDAAO) and prefrontal cortex lysates (as a negative control) by performing immunoprecipitation experiments. Western blot analysis showed that hDAAO was successfully isolated from the cerebellum lysate, but not from that of the prefrontal cortex (Figure 4A, left), the protein being expressed at very low levels in the latter brain region (Verrall et al., 2007; Sacchi et al., 2008). The Pro-Q Diamond gel staining following SDS-PAGE revealed that, in the cerebellum, the flavoenzyme is phosphorylated, although only to a low extent: a faint, positive 40-kDa band corresponding to phosphorylated hDAAO was observed in the immunoprecipitated sample from cerebellum whereas it was absent in the negative controls (Figure 4A, right).

We then performed *in vitro* phosphorylation experiments using fixed amounts of hDAAO (0.17 nmoles/reaction) and three commercial kinases: PKA (0.009 U/reaction), PKC-α (0.001 U/reaction), or PKC-ε (0.0005 U/reaction). Positive controls were set up using recombinant cAMP-responsive element-binding protein 1 (CREB1) or histone H1 in place of hDAAO. The analysis of PKA-treated samples showed a signal stained by the Pro-Q Diamond dye corresponding to the 59-kDa band of the positive control GST-tagged CREB1, whose intensity increased with the time of incubation, while hDAAO was not modified by the kinase (Figure 4B, top). Similar results were obtained for PKC-α- and PKC-ε-treated samples: a band corresponding to the histone H1 was evident upon staining for phosphoproteins while hDAAO was not detected (not shown). These findings suggest it is unlikely that PKA and PKC are involved in phosphorylation of the human flavoenzyme.

## Discussion

During the last decade, a relevant physiological role was highlighted for hDAAO in the central nervous system: in several areas, it is responsible for catabolizing the key endogenous NMDAr coagonist D-Ser (Sacchi et al., 2012; Murtas et al., 2017; Pollegioni et al., 2018). The flavoenzyme is therefore indirectly involved in modulating the activation state of these receptors and plays a role in NMDAr signaling pathway impairments, which are known to occur in acute and chronic neurological diseases and are often due to an imbalanced D-Ser metabolism (Collingridge et al., 2013; Paul and de Belleroche, 2014; Guercio and Panizzutti, 2018; Kondori eta l., 2018; Uno and Coyle, 2019; Ploux et al., 2020).

In this context, structural and functional studies performed on recombinant hDAAO unveiled several mechanisms that would modulate the enzyme’s properties (see Introduction). Here, we investigated the modulation of hDAAO properties by post-translational modification(s), as known for SR, the D-Ser biosynthetic enzyme (Mustafa et al., 2007; Raboni et al., 2019). Previous data indicated that, in astrocytes, NO inhibits SR and enhances hDAAO activity, boosting degradation of the neuromodulator (Shoji et al., 2006). S-nitrosylation was recognized as a reversible mechanism of allosteric regulation in several proteins, affecting their functioning, protein-protein interaction, receptor activation, and channel gating (Hess et al., 2005), which could be relevant in pathological conditions (Cho et al., 2009; Foster et al., 2009; Picón-Pagès et al., 2019). Selective protein S-nitrosylation has been linked to the regulation of NMDA and AMPA receptor activity, the surface expression of AMPA receptors, and d-serine production (Mustafa et al., 2007; Choi et al., 2000; Selvakumar et al., 2009; Selvakumar et al., 2013). The chemical modification of cysteine residues can result from different processes: the direct reaction with NO followed by oxidation (Hess et al., 2005) and the transnitrosylation either by S-nitrosylated proteins (Nakamura and Lipton, 2013) or by S-nitrosylated peptides, (GSNO) being among them (Broniowska et al., 2013). Although we know very little about selectivity for protein cysteine modification, it has been reported that cysteines located in surface-accessible areas with charged amino acids in the vicinity are vulnerable to nitrosylation (Gould et al., 2013). Here, we predicted the highly conserved Cys18 and Cys264 as putative nitrosylation targets as they are located on the surface of hDAAO and surrounded by residues that might stabilize nitrosothiols once formed (Figure 1). Experimental evidence supports this structure-based observation: under native conditions, both the holoenzyme and the apoprotein form of hDAAO possess two solvent-accessible cysteines. hDAAO modification upon *in vitro* nitrosylation by GSNO was confirmed by the sensitive fluorescent switch assay (Figure 2B) and demonstrated a partial, but significant inactivation of the enzyme (Figure 2C). Accordingly, we reported decreased hDAAO activity in U87 cells ectopically expressing the flavoenzyme treated with two different NO donors (Figure 2A). Additional *in vitro* studies showed that the inactivation effect was prevented by an excess of exogenous FAD (Figure 2C). As nitrosylation does not affect the apoprotein-FAD binding equilibrium (Table 3), the observed effect is due to the easier reaction of GSNO with the cysteines of the apoprotein of hDAAO, a form which is known to possess a “relaxed” tertiary structure characterized by greater exposure of hydrophobic surfaces and increased sensitivity to chemical and thermal unfolding (Caldinelli et al., 2009). Notably, S-nitrosylation of human SR affected its functionality by stabilizing an open, less-active conformation of the enzyme (Marchesani et al., 2020). Thus, this reversible modification might represent a common mechanism for simultaneously controlling both D-Ser metabolic enzymes in different and neighboring cells. In the brain, hDAAO is primarily expressed in the cerebellum astrocytes and Bergmann glia (Verrall et al., 2007). Notably, increased NO levels have been observed during reactive astrogliosis (a condition associated to a wide range of abnormalities and pathologies of the central nervous system) due to the induction of inducible nitric oxide synthase production in “activated” glial cells (Endoh et al., 1994). It is tempting to speculate that increased NO levels in reactive activated glia might lead to hDAAO modification: further investigations are needed to substantiate this hypothesis.

A second post-translational modification of cysteine residues is represented by S-sulfhydration, converting the–SH group of cysteines to a reactive persulfide or–SSH group (Paul and Snyder, 2015; Zhang et al., 2017). Sulfhydration is a highly prevalent modification: a variety of key proteins in different cellular pathways are modified by H_2_S, this affecting crucial cellular processes, including survival/death, differentiation, proliferation, metabolism, mitochondrial bioenergetics/biogenesis, endoplasmic reticulum stress, inflammation, and oxidative stress (Mustafa et al., 2009; Paul and Snyder, 2012). Here, we observed that the hydrogen sulfide donor NaHS apparently failed in covalently modifying recombinant hDAAO (Figure 3B) although it increased hDAAO activity in U87 cells ectopically expressing the flavoenzyme (Figure 3A) and stabilized the enzyme at a low concentration of FAD (Figure 3C) by inducing a tighter interaction of the apoprotein with FAD (Table 3). This result suggests that NaHS induced stabilization of a protein conformation with the highest avidity for FAD, which explains both the increased stability over time and the highest activity (Figure 3A). Considering that in the mammalian brain endogenous H_2_S concentrations range between 50 and 160 μM (Abe and Kimura, 1996; Wang, 2003), that it can be generated from d-cysteine by the coupled reaction of hDAAO and 3-mercaptopyruvate sulfurtransferase (Shibuya et al., 2013), and that an initial protein S-nitrosylation event can potentially promote the formation of a more enduring sulfhydration reaction, the gasotransmitter-mediated modulation of hDAAO activity might be relevant under specific conditions. Moreover, another enzyme responsible for endogenous H_2_S production in the brain, i.e., cystathionine *β*-synthase (CBS), is predominantly localized in Bergmann glia and astrocytes (Enokido et al., 2005). CBS expression is reduced upon inflammatory activation of astrocytes (Guo et al., 2012) while it is upregulated in acute ischemic conditions (Chan et al., 2015). Once more, we might assume that the consequent variations in H_2_S levels could differently affect hDAAO functionality.

Phosphorylation represents another post-translational modification common to the enzymes involved in metabolizing the neuromodulator. In murine SR, phosphorylation takes place at multiple sites, depending on the enzyme’s cellular localization, but mainly at Thr71, and was shown either to increase or to decrease SR activity (Balan et al., 2009; Foltyn et al., 2010; Vargas-Lopes et al., 2011). The activation of PKC and the resulting SR phosphorylation reduced D-Ser levels in astrocytes and neuronal cultures and in rat frontal cortex (Vargas-Lopes et al., 2011). Here, we observed that hDAAO, which is highly expressed in the cerebellum, is phosphorylated, too, although only to a low extent (Figure 4). However, although in silico analysis suggested that hDAAO is modified by kinases belonging to the serine/threonine family, *in vitro* analyses failed to demonstrate high-stoichiometric phosphorylation by PKA or PKC. Further studies are needed to identify the kinase(s) involved and the physiological mechanism related to this post-translational modification. This is a relevant issue since recent studies reported: i) a relationship between the activation of extracellular signal regulated kinase (ERK) pathway in diabetic retina and DAAO level (Jiang et al., 2021); ii) how an increased d-serine level augmented the activity of nNOS (increasing NO) and thus of the phosphorylated form of GluN1 subunit of NMDAr by PKC in a mouse model of neuropathic pain (Choi et al., 2019); iii) how a decreased d-serine level increased the phosphorylated degree of Akt in motoneurons of SOD1^G93A^ mice (Wang et al., 2017) or inhibited the phosphorylation level of GSK3β, ERK1/2 and CREB in primary cultured neural stem cells (Huang et al., 2012).

Two main limitations of our study are the need to i) verify the suggested modification of Cys18 and Cys264 in different tissues as well as in U87 cells ectopically expressing hDAAO subjected to different stimuli, and ii) confirm hDAAO phosphorylation in the cerebellum of a statistically significant number of human subjects.

In conclusion, this investigation elucidated novel molecular mechanisms regulating hDAAO functionality, which, intriguingly, are common to SR, i.e., the other enzyme involved in D-Ser metabolism. Based on these findings we argued that evolution likely adopted complicated regulatory strategies to keep the flavoenzyme under stringent functional control (Sacchi et al., 2012; Murtas et al., 2017) and, ultimately, to modulate D-Ser levels in human brain under physiological and pathological conditions.

## Data Availability

The raw data supporting the conclusions of this article will be made available by the authors, without undue reservation.

## Abbreviations

BSA (bovine serum albumin), CBS (cystathionine β-synthase), CREB1 (cAMP-responsive element-binding protein 1), DAAO (D-amino acid oxidase), D-Ser (D-serine), DTNB (5,5′-dithiobis-(2-nitrobenzoic acid)), ERK (extracellular signal regulated kinase), FAD (flavin adenine dinucleotide), GSH (reduced glutathione), GSNO (S-nitrosoglutathione), GSSG (oxidized glutathione), hDAAO (human DAAO), IP (immunoprecipitation), MMTS (methyl methanethiosulfonate), NMDAr (N-methyl-D-aspartate receptor, NO (nitric oxide), NOS (nitric oxide synthase), PKA (cAMP-dependent protein kinase), PKC (protein kinase C), SAS (solvent accessible surface area), SDS-PAGE (sodium dodecyl sulfate–polyacrylamide gel electrophoresis), SNO-Cys (S-nitrosylated cysteine), SR (serine racemase)
